# Effect of fieldwork-friendly coffee blender-based extraction methods and leaf tissue storage on the transcriptome of non-model plants

**DOI:** 10.1007/s10265-025-01624-w

**Published:** 2025-03-07

**Authors:** Shine-Undarga Dagva, Josephine Galipon

**Affiliations:** 1 Graduate School of Science and Engineering, Yamagata University, Yonezawa, Yamagata Japan; 2 Institute for Advanced Biosciences, Keio University, Tsuruoka, Yamagata Japan; 3 Graduate School of Media and Governance, Keio University, Fujisawa, Kanagawa Japan

**Keywords:** De novo assembly, Fieldwork, Non-model Plant, RNA extraction, Tissue lysis, Transcriptome

## Abstract

The adaptation of plants to environmental conditions involves a transcriptional response. “Field transcriptomics” is an emerging concept for studying plants in their natural habitat. However, this term includes studies in which cold storage was possible until further processing in a laboratory. Previous studies proposing onsite RNA extraction methods are limited to descriptions of RNA purity, quantity, and quality, and lack a thorough evaluation of transcriptome quality, and transcriptomic evaluations of RNA storage solutions in plants are, to our knowledge, only available for periods of less than a day. This issue is critical for studying plants in geographically difficult-to-access regions, where keeping the cold chain is unrealistic. In this study, the transcriptome of the non-model plant *Helonias orientalis* (order: Liliales) was evaluated before and after storage of the leaf tissue for one and fourteen days at 25 °C in RNA*later* and TRIzol, respectively. Additionally, field-friendly protocols were similarly evaluated for onsite plant RNA extraction at ambient temperature with lightweight equipment that can run on a portable generator, including a guanidine isothiocyanate-free protocol that is compatible with the polyphenol-rich wild strawberry *Fragaria vesca*. The quality of the transcriptome assembly after 1-day storage and our optimized onsite methods had similar results to that of the state-of-the-art. However, in terms of differential expression analysis, onsite extraction methods performed better overall than the stored tissue samples. We expect that our onsite RNA extraction methods will provide valuable insights into the transcriptional regulation of plants in areas where research equipment is difficult to access.

**Supplementary Information:**

The online version contains supplementary material available at 10.1007/s10265-025-01624-w.

## Introduction

Plants adapt to various environmental conditions such as drought, flood, and temperature change by inducing changes in metabolism, which are instigated by changes in gene expression (Mareri et al. 2022). This is generally studied by measuring their transcriptomic and proteomic profiles (Haider et al. 2013). Proteomics relies on the mass spectrometry of digested peptides, meaning that prior information about the coding regions is required. De novo proteomics without any reference remains challenging, especially given the complexity of plants (Jorrin-Novo et al. 2015; Zmeinko et al. 2022). Unlike protein, RNA can be amplified by PCR after reverse transcription especially, meaning that the signal can be amplified from small amounts of starting material. Therefore, transcriptomics is the method of choice for studying gene expression in non-model organisms, as it provides an overview of gene expression levels at a given time in a particular environmental condition, even in the absence of prior knowledge of the organism’s genome.

Field transcriptomics is a subfield of transcriptomics that focuses on studying how plants react to the complex conditions found in their natural environments. (Ichihashi et al. 2018; Ma et al. 2017; Satake et al. 2019; Sato et al. 2019). RNA expression is easily influenced by external stimuli and RNA itself is sensitive to degradation, so flash-freezing in liquid nitrogen and keeping the cold chain is critical. To our knowledge, field transcriptomics research relies on liquid nitrogen or RNA preservation solutions, and the samples are kept cooled or frozen until RNA extraction. Hence, most field transcriptomics research is done at sampling sites located within a few hours of an equipped laboratory (Ichihashi et al. 2018; Ma et al. 2017; Satake et al. 2019; Sato et al. 2019). Depending on the fieldwork location, transportation of liquid nitrogen may be dangerous, especially in rough outdoor terrain. Keeping the cold chain also requires a steady supply of electricity, which is difficult to achieve in remote locations such as deserts, mountains, and rainforests, which may be days away from any properly equipped laboratory.

Furthermore, plant RNA extraction and storage methods frequently stop short of evaluating the transcriptome, instead providing measurements of RNA quantity, quality, and purity (Gambino et al. 2008; Mommaerts et al. 2015; Yu et al. 2012). While those parameters are of course a very important parameter to enable successful transcriptomics, they are not a guarantee of success. For example, popular RNA quality measurements such as the RNA Integrity Number (RIN) were developed exclusively based on mammalian data, and additional ribosomal RNA (rRNA) bands in plants may result in RIN number underestimation to various degrees in lants (Kim et al. 2014). Furthermore, different transcripts degrade at different rates, implying that it is possible for two samples with a similar RIN to result in different levels of completeness at the transcriptome assembly level (Gallego Romero et al. 2014; Wang et al. 2016).

Therefore, in this study, we aimed to evaluate fieldwork-friendly plant tissue storage and onsite plant RNA extraction methods not only from the point of view of RNA quality, quantity, and purity, but also from the perspective of transcriptome assembly and the quantification of gene expression, using a non-model plant. *Helonias orientalis* (Thunb.) N. Tanaka (order Liliales, family Melanthiaceae) is a perennial plant that is broadly distributed in Japan and known for the formation of adventitious plantlets at the leaf tip as a mode of asexual reproduction (Kato et al. 1972; Kawano et al. 1980; Miyazaki et al. 1999). The genome size is estimated to be 3.1 Gb for a karyotype of 2n = 34 (Pellicer et al. 2014), but Liliales are understudied at the molecular biology level, with no genome sequence available for the entire order (Marks et al. 2021). To assess plant tissue preservation methods on this type of non-model plant, the two different preservation solutions presented below were tested at two different storage periods (1 day and 14 days at ambient temperature) on *H. orientalis* leaf tissue. RNA*later* (Invitrogen^™^ RNA*later*^™^ Stabilization Solution) is one of the most widely used cell and tissue preservation reagents. It stabilizes the RNA and protein contained in soft tissue and cell culture samples, but its effectiveness is not guaranteed by the manufacturer for plants, insects, and soil samples, meaning that prior testing on one’s type of target sample is recommended. A high concentration of quaternary ammonium sulfate and cesium sulfate in the reagent protects RNA and proteins rom degradation by denaturing RNases, DNases, and proteases in cells (Bennike et al. 2016). The effect is dependent on the solution’s ability to permeate the membrane of intact cells, and works better for thin pieces of tissue (Salehi et al. 2014). It remains unclear how the plant cell wall affects the permeability of these chemicals, and how thin the sample tissue should be cut. So far, only one paper evaluated the effect of storage for 12 h at ambient temperature in RNA*later* on the transcriptome of *Arabidiopsis thaliana* (Kruse et al. 2017). Our study is the first to investigate the effect of RNA*later* on the transcriptome of a non-model plant, and for over a longer storage period.

TRIzol (Invitrogen^™^ TRIzol^™^ Reagent) is a well-known RNA, DNA, and protein extraction reagent that is designed based on the single-step RNA isolation method developed by Chomczynski and Sacchi (1987). TRIzol is commonly used for the isolation of RNA in human, animal, plant, yeast, or bacterial samples. Lysed samples may be stored in TRIzol at − 80 °C or − 20 °C, but no similar reports for plant tissue storage could be found (Eikmans et al. 2013; Ma et al. 2010; Pérez‐Portela et al. 2013). In this study, to investigate the usability of these solutions for the preservation of plant tissue in the context of fieldwork in remote areas, plant tissue was stored at room temperature for 1 and 14 days in TRIzol and RNA*later*, respectively, and subjected to transcriptome analysis.

Our results show that RNA is degraded after two weeks of storage using either condition, highlighting the need for onsite RNA extraction in the context of lengthy fieldwork expeditions. In the second part of this study, we compared field-friendly protocols for coffee blender-based plant RNA extraction, which is compatible with lightweight equipment that can be run on a gasoline or solar-based generator, using the leaves of Japanese hyacinth *H. orientalis*.

For RNA and DNA extraction from plants, grinding the plant material in liquid nitrogen using a mortar and pestle is considered to be the state-of-the-art. After grinding, the sample is resuspended in suitable solutions such as TRIzol or CTAB (Cetyltrimethylammonium Bromide) extraction buffer (Tan et al. 2009). In this study, we used different combinations of TRIzol and/or CTAB-based extraction buffers, and evaluated the suitability of an electric coffee bean blender as an alternative to liquid nitrogen for tissue disruption. Transcriptomics results for three different field-friendly RNA extraction protocols are presented and discussed.

Additionally, this research is the first to sequence the transcriptome of *H. orientalis*. This study shows that plant RNA cannot be stored in TRIzol nor RNA*later* for 2 weeks at room temperature, demonstrating the need for onsite extraction methods. Finally, the coffee blender-based onsite extraction methods tested show promising results, albeit with some reservations depending on the purpose of the study.

## Materials and methods

*Plant materials*: Individuals of *H. orientalis* were transplanted into pots from their natural habitat in Mount Takadate (Tsuruoka City, Yamagata Prefecture, Japan) with the official signed permission from the Shonai Forest Office in May 2018, and cared for at the Institute for Advanced Biosciences (Keio University, Tsuruoka City, Yamagata Prefecture, Japan) until RNA extraction in 2021–2022.

*Solutions and reagents*: Liquid Nitrogen, Invitrogen TRIzol^®^ reagent (Thermo Fisher Scientific Inc.), CTAB Extraction Buffer (2% CTAB: Hexadecyltrimethylammonium Bromide, 100 mM Tris–HCl pH 8, 25 mM EDTA pH 8, 2 M NaCl, 2.5% PVP K-30, 1.5% β-mercaptoethanol (β-mercaptoethanol is added right before the extraction)), Invitrogen RNA*later*^™^ Solution (Thermo Fisher Scientific Inc.), Chloroform (Wako Pure Chemical Industries, Ltd), 2-Propanol (Isopropyl Alcohol) (Wako Pure Chemical Industries, Ltd), 75% Ethanol (Wako Pure Chemical Industries, Ltd), MilliQ water, 7.5 M ammonium acetate NH4OAc, and Recombinant DNase I (Takara Bio Inc.): 10 × DNase I Buffer, rDnase I.

**Fig. 1:**
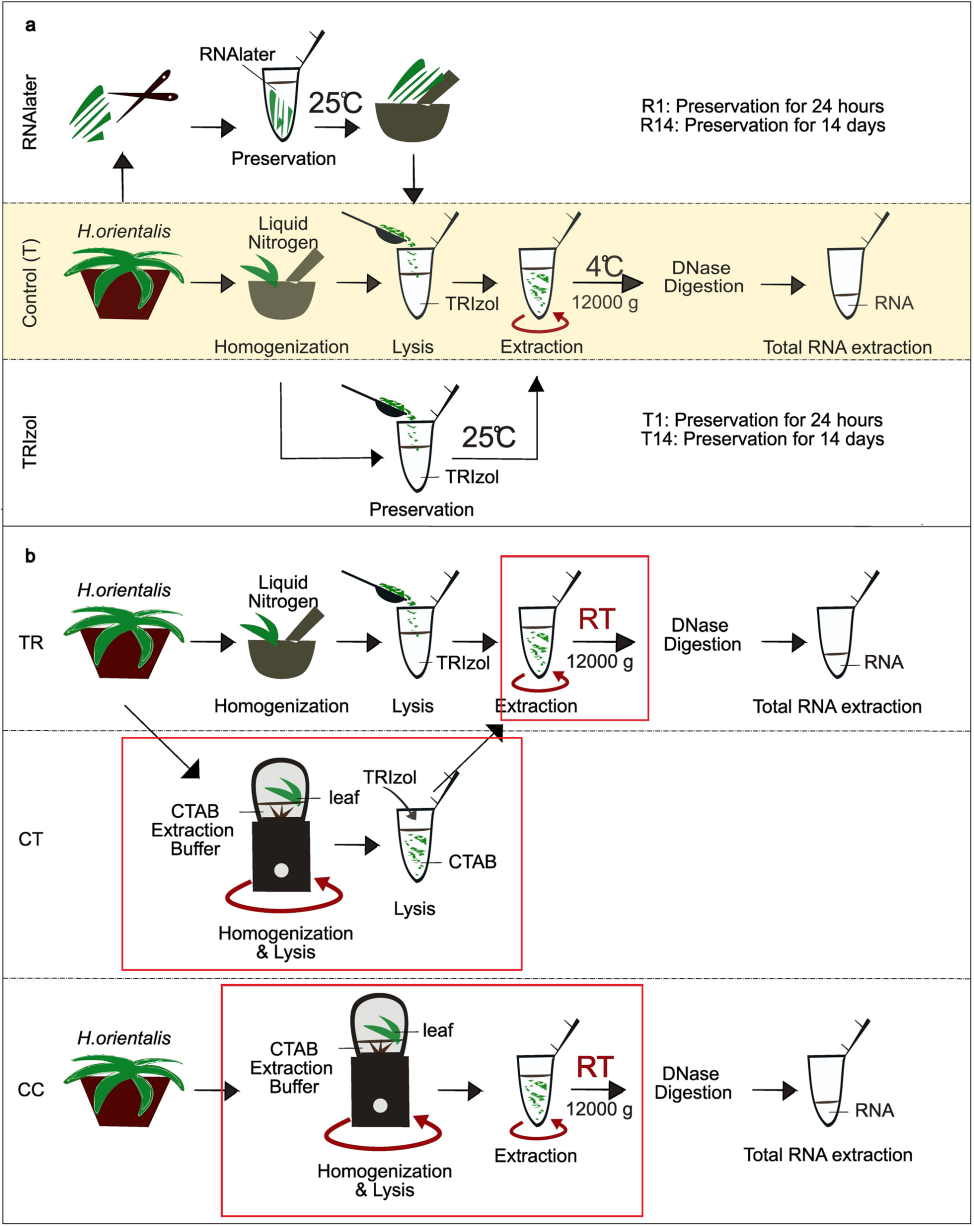
Schematic illustration of the protocols used for tissue storage and RNA extraction. **a** RNA storage methods. **b** RNA field-friendly onsite extraction methods. R1: RNA*later* preservation for 1 day at room temperature, R14: RNA*later* preservation for 14 days at room temperature, Control (T): Control using TRIzol method, T1: TRIzol preservation for 1 day at room temperature, T14: TRIzol preservation for 14 days at room temperature, TR: TRIzol at room temperature onsite extraction method, CT: CTAB in coffee bean blender, then TRIzol onsite extraction method, CC: CTAB in coffee bean blender onsite extraction method, RT: room temperature. Following total RNA extraction, mRNA poly(A) enrichment, sequencing, and transcriptome analysis were conducted. Red lines show the changes that were made for the onsite extraction. Please see the detailed protocols in the Supplementary Information

*Total RNA extraction*: Two tissue preservation methods with two different periods and three different methods of onsite RNA extraction protocols using portable equipment described in Table 1 were compared with the state-of-the-art method by TRIzol reagent (Fig. 1, SUP 1, Fig. S1, Fig. S2). After nucleic acid extraction, all the samples were digested by recombinant DNase I (Takara Bio Inc.) according to the manufacturer’s instructions with small modifications using Eppendorf MiniSpin^®^ plus. Please see detailed explanations in the Supplementary Information. RNA yield was calculated from the plant tissue weight and RNA quantity that is obtained by Nanodrop ND-1000 Spectrophotometer while RNA integrity was verified using an Agilent 2100 Bioanalyzer. The statistical analysis was calculated by ANOVA post hoc Tukey Honestly Significant Difference (HSD) test (https://astatsa.com/OneWay_Anova_with_TukeyHSD/ Accessed on 31 May 2024).

**Table 1:** Portable equipment used for onsite extraction methods

	Equipment	Manufacturer	Explanation
1	Vitantonio Mini Bottle Blender VBL-6 (in this paper, it is mentioned as a coffee blender, Fig. S1)	Sanyei Corporation, Tokyo, Japan	Used as an alternative to the liquid nitrogen in the mortar and pestle step
2	Eppendorf MiniSpin^®^ plus	Eppendorf SE, Hamburg, Germany	Used as an alternative to more bulky refrigerated centrifuges

*mRNA library preparation and sequencing*: to obtain enough RNA for transcriptomics, all available replicates for each sample were merged. 300 bp fragment size strand-specific libraries were produced by NEBNext^®^ Ultra^™^ II Directional RNA Library Prep Kit after mRNA was isolated by NEBNext^®^ Poly(A) mRNA Magnetic Isolation Module. Samples were sequenced in 2 × 150 bp paired-end mode using the Novaseq 6000 Sequencing System (Illumina Inc. San Diego, USA), resulting in more than 25 million raw reads for onsite protocols and 35 million raw reads for storage protocols.

*Transcriptome analysis*: In order to rule out any effect of variable sequencing depth on the quality of the transcriptome assembly, the total raw data was set to approximately 8 Gb (right reads: 4 Gb, left reads: 4 Gb) for each sample. For samples which had more than 8 Gb data, the data were downsampled by random selection using seqtk (Li 2012). Quality control of sequencing files was done by FastQC (Andrews et al. 2010). Clean paired reads were obtained by trimming the raw reads at the minimum Phred score of Q = 20, adapters were trimmed with a minimum overlap (stringency) of 6 bp, followed by removal of reads shorter than 100 bp, as well as non-paired reads, using Trim Galore version 0.6.7 (Cutadapt version 4.2) (Felix et al. 2023 and Table S1). Clean paired reads for each sample were assembled separately with Trinity v.2.11.0 using default parameters (kmer size 25 bp). To compare the transcriptome quality for each protocol, quality assessment was done by 1. checking the transcriptome statistics (SeqKit v.2.3.0), 2. mapping the reads back to the assembly (Bowtie2 version 2.4.4), and 3. checking transcriptome completeness by BUSCO version 5.4.4 (Benchmarking Universal Single-Copy Orthologs) (Langmead et al. 2019; Manni et al. 2021; Shen et al. 2016). BUSCO was run on the monocotyledon-specific database (liliopsida_odb10). To quantify individual transcripts, the samples for each experiment (“onsite methods T, TR, CT, CC”, “RNAlater storage T, R1, R14”, and “TRIzol storage T, T1, T14”) were combined to create three common de novo transcriptome references (Trinity v.2.11.0 using default parameters). Transcriptome quantification was done by running the pseudoaligner Salmon version 1.2.0 (Patro et al. 2017). The threshold for differentially expressed genes was a twofold change in the TPM + 1 value relative to the control. Open reading frames (ORFs) in the transcripts were predicted by Transdecoder 5.5.0 (Haas et al. 2013). These ORFs were then evaluated by InterProScan-5.62–94.0 (databases: CDD-3.20, Coils-2.2.1, Gene3D-4.3.0, Hamap-2023_01, MobiDBLite-2.0, NCBIfam-11.0, PANTHER-17.0, Pfam-35.0, PIRSF-3.10, PRINTS-42.0, SFLD-4, SMART-9.0, SUPERFAMILY-1.75) to obtain Gene Ontology IDs for the transcripts (Jones et al. 2014). To understand the functional meaning of the differentially expressed genes (twofold change), Gene Ontology enrichment analysis was done with BiNGO on the Cytoscape platform (version 3.10.0). Annotation files were custom-made with a Python script provided by Duarte et al. (2021) and adjusted to the format introduced in the BiNGO customization tutorial (Duarte et al. 2021; Shannon et al. 2003).

*RT-qPCR validation*: The transcripts with a GO annotation were ranked by order of the coverage (TPM) in the T control, and separated into three groups of equal size. A total of approximately 100 transcripts was randomly selected equally among these groups. Then, primers targeting the open reading frame (ORF) were designed for each of these transcripts using the command line version of Primer3. These primers were then subjected to a BLASTn search against the reference transcriptome. Primer pairs for which the 3’-ends matched any other location were discarded, resulting in the retention of 18 gene-specific primer sets (Table S3). cDNA for all replicates were obtained using the PrimeScript 1st strand cDNA Synthesis Kit, with poly-dT primers (Takara). PCR was carried out with the Ex Taq Hot Start DNA Polymerase (Takara) (annealing temperature: 55 ºC). DNA was resolved on a 4% agarose (Promega, LMP, Preparative Grade for Small Fragments), 1X TBE, 100 V, 40 min, and after-staining with Midori Green Xtra (Nippon Genetics). The genes with a single band of the correct size were selected for further analysis by using 2-step RT-qPCR with melting curve, using SYBR Green and internal ROX dye calibration (Toyobo) on a ViiA 7 Real-Time PCR System (Applied Biosystems). The single band obtained in the initial PCR check (Fig. 3a) was purified, its quantity measured by Qubit 3.0 fluorometer (dsDNA broad range kit, Thermo Fisher Scientific), and used as a standard dilution series for gene-specific quantification.

## Results

### RNA yield and quality for tissue preservation and onsite extraction methods

First, to assess the efficacy of tissue preservation methods, total RNA extraction was performed after storing *H. orientalis* leaf tissue in either RNA*later* or TRIzol for 1 and 14 days at 25 °C. For RNA*later*, the leaf was cut into 1 mm-wide strips using sterilized scissors. For TRIzol, the leaf tissue was homogenized in liquid nitrogen using a mortar and pestle (Fig. 1). Since the RNA*later* and TRIzol experiments were done on different dates, the results were compared with their respective controls, which were the standard TRIzol extraction procedure conducted immediately after grinding in liquid nitrogen (Control (T)). The RNA quality was evaluated using a proprietary algorithm from Agilent 2100 Bioanalyzer known as the RNA Integrity Number (RIN) (cf. Material and methods).

**Fig. 2:**
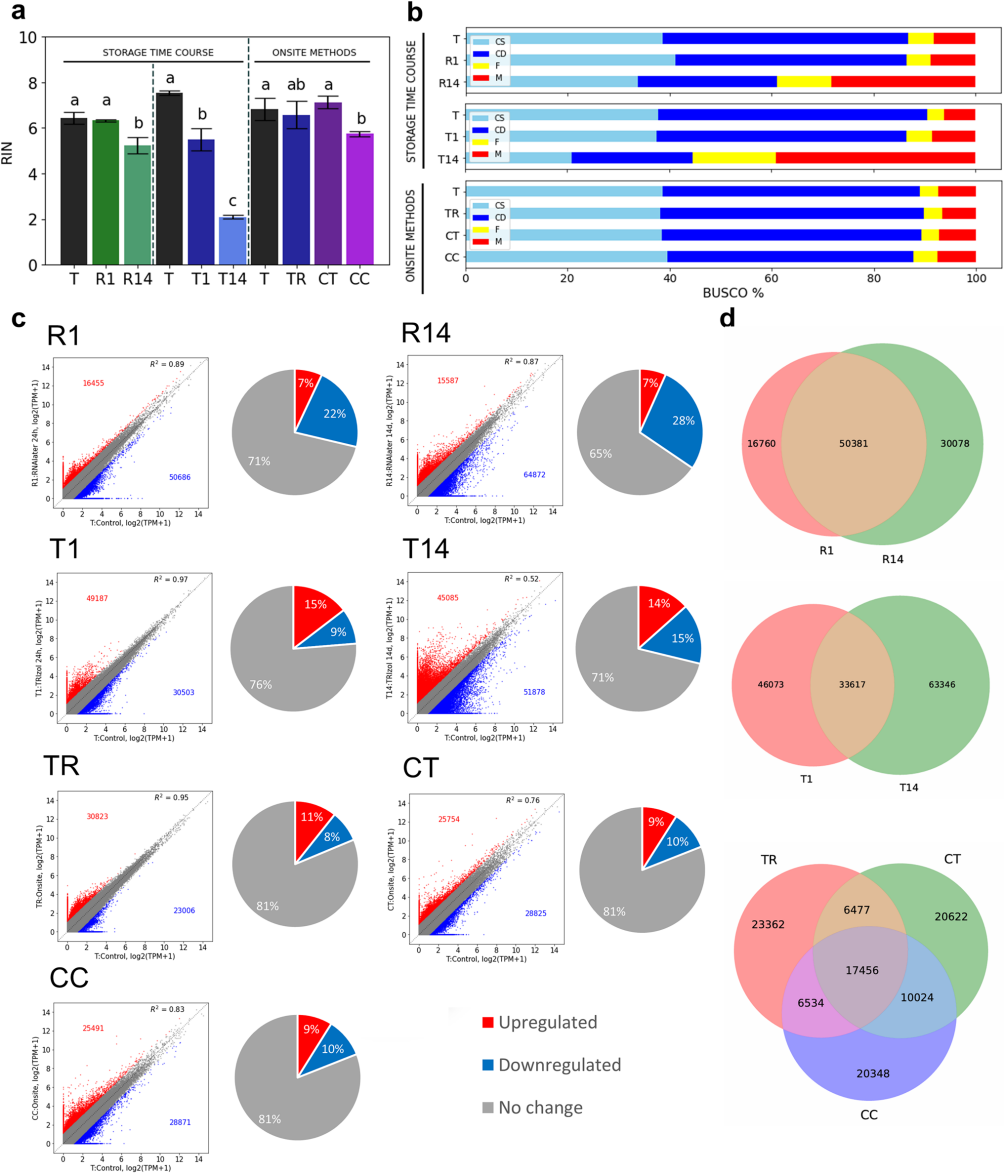
Results of the optimized extraction protocols for preservation and field-friendly extraction methods. **a** Bar plot showing the mean RNA quality (RIN) obtained with the Agilent 2100 Bioanalyzer and Agilent RNA 6000 Nano Kit for storage time course methods (*n* = 3), and onsite extraction protocols (*n* = 4). Means with different subscripts differ significantly (ANOVA post hoc Tukey HSD test: *P* < 0.05). The data is the same as in Table 2. **b** Transcriptome completeness by Benchmarking Universal Single-Copy Orthologs (BUSCO 5.4.4 liliopsida_odb10 (*n* = 3,236)). **c** Results of differential expression compared to their respective control “T”. Differential expressed transcripts with a twofold change are highlighted in red (upregulated) and blue (downregulated), respectively. **d** Venn diagram showing the overlap of total DEGs (up- and downregulated) overlapping between samples

The preservation of *H. orientalis* plant leaf tissue for 1 and 14 days in RNA*later* (R1 and R14) or TRIzol (T1 and T14) did not affect total RNA yield (Table 2). The 1 day RNA*later* preservation condition showed no effect on RNA quality (RIN), but the RIN dropped from 6.4 to 5.2 after 14 days of preservation (*P* < 0.05, Table 2, Fig. 2a and Fig. S3). In contrast, preservation in TRIzol showed faster RNA degradation from RIN 7.7–5.5 after 1 day, and to 2.1 after 14 days (*P* < 0.05).

**Table 2:** Average RNA yield, purity, and quality of the extracted samples for storage time course methods (*n* = 3), and onsite extraction protocols (*n* = 4)

Methods	RNA yield (µg/g)	260/280	260/230	RIN
Storage time course				
T	205.7 ± 37.5 a	1.85 ± 0.03 a	1.60 ± 0.07 a	6.4 ± 0.2 a
R1	210.2 ± 52.0 a	1.89 ± 0.01 a	1.60 ± 0.03 a	6.3 ± 0.05 a
R14	155.7 ± 15.7 a	1.65 ± 0.02 b	0.71 ± 0.25 b	5.2 ± 0.4 b
T	161.9 ± 53.8 a	1.68 ± 0.03 ab	1.06 ± 0.03 b	7.5 ± 0.1 a
T1	115.9 ± 11.9 a	1.66 ± 0.06 b	0.99 ± 0.04 b	5.5 ± 0.5 b
T14	124.7 ± 19.8 a	1.82 ± 0.05 a	1.60 ± 0.17 a	2.1 ± 0.01 c
Onsite methods				
T	70.0 ± 13.6 b	1.83 ± 0.04 ab	1.73 ± 0.21 ab	6.8 ± 0.5 a
TR	91.5 ± 18.7 b	1.87 ± 0.02 a	1.99 ± 0.18 a	6.6 ± 0.6 ab
CT	241.8 ± 24.0 a	1.81 ± 0.01 b	1.65 ± 0.05 ab	7.1 ± 0.3 a
CC	117.1 ± 24.5 b	1.79 ± 0.02 b	1.59 ± 0.07 b	5.8 ± 0.1 b

Means with different subscripts differ significantly (ANOVA post hoc Tukey HSD test: *P* < 0.05). “T” protocol is the TRIzol control for each experiment. ± shows the standard deviation. These RIN values were used to produce the bar plot in Fig. 2

Next, to assess field-friendly methods using portable equipment for distant natural habitats of non-model plants, three different onsite methods, namely TR: TRIzol room temperature, CT: CTAB in blender + TRIzol and CC: CTAB in blender, were carried out (Fig. 1). All three methods were performed with a portable, non-refrigerated centrifuge, while homogenization steps in CT and CC methods were performed by using CTAB extraction buffer in a coffee bean blender (equipment described in Table 1, Fig. S1, Fig. S2). After coffee-blender homogenization, TRIzol was added in the CT method, and any remaining steps were done by following the TRIzol extraction method at room temperature. On the other hand, the CC method was carried out by the CTAB extraction method as in Gambino et al. (2008), but without lithium chloride precipitation and at room temperature. The results were compared with the state-of-art method (T) and RNA quality was evaluated by RIN.

The CT method showed significantly higher RNA yield (*P* < 0.05, Table 2). The field-friendly TR and CT protocols showed RIN of 6.6 and 7.1 respectively, which were not significantly different from the TRIzol control (RIN 6.8), while the CC protocol showed significant RNA degradation (RIN 5.8, *P* < 0.05, Table 2, Fig. 2a and Fig. S3).

### *De novo* transcriptome assembly

The effect of storage (R1, R14, T1, and T14) and field-friendly protocols (TR, CT, and CC) on the transcriptome quality was evaluated. Since no reference transcriptome or genome is available for *H. orientalis*, we performed de novo assembly before transcript quantification. A recent evaluation of the effect of sequencing depth on de novo transcriptome assembly for the tea plant *Camellia sinensis*, the genome of which is similar in size to that of *H. orientalis* (~ 3 Gb), showed that 8 gigabases (Gb) data provided over 80% BUSCO completeness for that plant (embrophyta_odb9 (*n* = 1440)) (Li et al. 2019). Based on this result, we assume that 8 Gb sequencing data is sufficient for protocol evaluation.

The N50 statistic is the sequence length at which half of the assembled transcripts match or exceed this value (weighted median). Lower N50 values may indicate a fragmented assembly. The BUSCO (Benchmarking Universal Single-Copy Orthologs) tool compares a universal gene set with an input transcriptome data, to determine the proportion of ‘CS: Complete Single’, ‘CD: Complete Duplicated’, ‘F: fragmented’ and ‘M: Missing’. Here, we estimated the completeness of the assembly by comparing to the monocotyledon-specific universal gene set (cf. Methods). Another commonly used method to assess the quality of a transcriptome assembly is to attempt to map the original pre-assembly reads to the newly assembled transcriptome; the percentage of mapped reads is indicative of how many reads in the original dataset contributed to the assembly. The percentage of reads mapping back to the assembly was similar across all samples, indicating successful assembly (Table 3).

**Table 3:** Statistics for the *H. orientalis* transcriptome assemblies of storage and onsite extraction protocols

Methods	RIN*	N50 (nt)	No. transcripts	Min Length (nt)	Max Length (nt)	Reads mapped** (%)
T	6.9	1,302	166,503	180	13,398	99.40
R1	7	1,411	106,102	191	14,947	99.61
R14	5.2	1,056	79,523	193	8,349	99.66
T	7.7	1,263	172,645	190	14,688	99.14
T1	7.1	1,134	177,842	187	10,077	99.27
T14	2.3	831	116,730	195	4,250	99.43
T	6.5	1,383	144,395	189	12,694	99.36
TR	6.7	1,379	160,288	186	13,647	99.41
CT	7.1	1,397	127,422	180	13,330	99.47
CC	5.2	1,346	129,535	185	9,839	99.45

^*^Total RNA quality of the representative sample of each protocol before mRNA isolation, and RNA sequencing

^**^Percentage of the reads mapped back to the assembly that was conducted by Bowtie2

The N50 length and BUSCO completeness suggest that storing plant tissue in RNA*later* or TRIzol for 1 day did not change the overall quality of the assembly. However, RNA*later* or TRIzol for 14 days resulted in less than 80% BUSCO completeness, as well as in a decrease in the N50 and total number of assembled transcripts, all of which are indicative of a fragmented assembly (Table 3 and Fig. 2b). Interestingly, R14 and T14 had very different RIN values (5.2 and 2.3, respectively), but resulted in similar evaluations.

The transcriptome assembly of onsite protocols was similarly evaluated. The results seem to show minimal differences between all onsite protocols (TR, CT, and CC) and the control, resulting in N50 values ranging from 1346 to 1397 nt, and BUSCO completeness scores of 87.7–89.8% (Table 3 and Fig. 2b). This is indicative of a satisfactory transcriptome assembly for fieldwork-compatible methods.

### Differential expression analysis and gene ontology enrichment analysis

To investigate the effect of tissue storage or onsite RNA extraction methods on gene expression, we conducted differential expression analysis and gene ontology enrichment analysis. At a twofold cut-off, the number of downregulated genes increased from Day 1 to Day 14 in both RNA*later* (from 22 to 28%) and TRIzol (from 9 to 15%), whereas the upregulated genes did not (Table 4 and Fig. 2c). The TRIzol method had double the percentage of upregulated genes (14 – 15%) compared to RNA*later* (7%) at all time points. On the other hand, the three onsite methods all showed similar numbers of differentially expressed genes (9 – 11% upregulated, 8 – 10% downregulated), totaling around 19% of the total consensus transcripts, which was overall better than any of the storage methods (Table 4 and Fig. 2c).

**Table 4:** The number of the differentially expressed genes by twofold cutoff when TPM + 1 values by SALMON were compared with their respective controls

Methods	Overall transcripts	Upregulated	Downregulated	R^2^
R1	233,650	16,455	50,686	0.89
R14	233,650	15,587	64,872	0.87
T1	336,154	49,187	30,503	0.97
T14	336,154	45,085	51,878	0.52
TR	287,088	30,823	23,006	0.95
CT	287,088	25,754	28,825	0.76
CC	287,088	25,491	28,871	0.83

The overall number of transcripts (common de novo transcriptome reference) was determined by running the assembly on the reads of all of the samples for each experiment using Trinity v.2.11.0 with default parameters

In terms of correlation score when comparing each protocol with its respective “T” control, TRIzol storage at Day 1 (T1) and TRIzol extraction at room temperature (TR) both correlated best with an R^2^ of 0.97 and 0.95, respectively (Table 4 and Fig. 2c). Next in the ranking were the R1, R14, and CC (0.89, 0.87, and 0.83, respectively), which were more correlated with their respective controls than either CT or T14 (0.76 and 0.52, respectively) (Fig. 2c).

Figure 2d shows the overlap between differentially expressed genes (DEGs) in each sample. Out of 233,650 transcripts (Table 4), DEGs unique to either R1 or R14 totalled 16,760 (7% of all transcripts; 25% of all DEGs in R1) and 30,078 (13% of all transcripts; 37% of all DEGs in R14), respectively. In contrast, out of 336,154 transcripts, DEGs unique to T1 and T14 totalled 46,073 (14% of all transcripts; 58% of all DEGs in T1) and 63,346 (19% of all transcripts; 65% of all DEGs in T14), respectively. These results indicate that the effect of storage in TRIzol and RNA*later* are profoundly different at transcript level, even after 1 day storage when the RIN and BUSCO scores did not significantly differ. In the case of onsite RNA extraction protocols, among 287,088 transcripts, the TR, CT, and CC protocols showed 23,362 (8% of all transcripts; 43% of all DEGs in TR), 20,622 (7% of all transcripts; 38% of all DEGs in CT), and 20,348 (7% of all transcripts; 37% of all DEGs in CC) unique DEGs, respectively, suggesting that the choice of onsite protocol is a critical factor.

To understand the biological significance of these differentially expressed genes, gene ontology enrichment was evaluated (Fig. S4). Storage in RNA*later* resulted in upregulation of photosynthesis at both time points (*P* < 1 × 10^–10^ and *P* < 1 × 10^–15^), and downregulation of the response to acid chemical, salt and water (*P* < 0.01), while storage in TRIzol resulted in reduced transcript abundance in intracellular protein transport (*P* < 0.01). Onsite extraction methods inevitably showed downregulation of translation (*P* < 1 × 10^–15^). Finally, CTAB-based protocols CT and CC resulted in an increase in the abundance of transcripts involved in photosynthesis (*P* < 1 × 10^–5^ for both), and carboxylic acid metabolic processes (*P* < 1 × 10^–10^ and *P* < 0.01, respectively), among others. These results confirm that the type of transcript affected is highly influenced by the method of storage or RNA extraction employed, suggesting that onsite RNA extraction methods should be properly evaluated and chosen carefully based on the purpose of the study. The relative fold-change and GO enrichment details for the DEGs which were assigned a GO are available as Table S2.

**Fig. 3:**
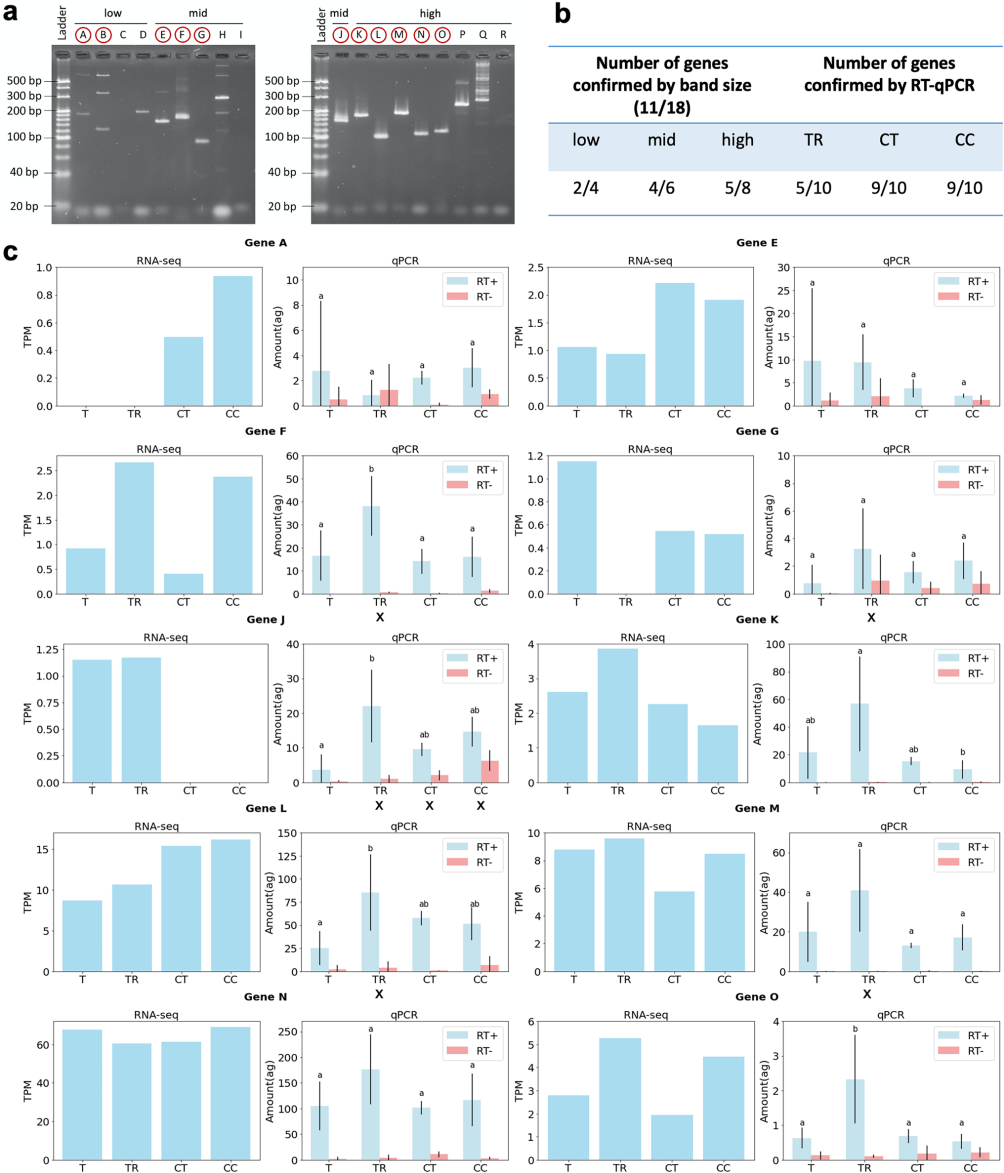
Cross-validation of the de novo transcriptome assembly and gene expression analysis by RT-qPCR. **a** Gel electrophoresis after PCR amplification of 18 candidate genes; the genes harboring a specific band of the correct size are circled in red (more information is available in Table S3). **b** Overview of the number of confirmed genes over the number of tested genes in each scenario. **c** Comparison of gene expression values obtained by RNA-seq and RT-qPCR (*n* = 4); X: the RNA-seq result was not confirmed by the RT-qPCR, the absence of an X indicates that the result was confirmed by RT-qPCR. RT + : Reverse Transcriptase positive sample, RT-: Reverse Transcriptase negative control. Gene E, originally randomly selected from “low”, was moved to the “mid” category after considering the sum of the TPM of its four transcript isoforms. Means with different subscripts differ significantly (ANOVA post hoc Tukey HSD test: *P* < 0.05)

To validate these results, transcripts with a GO annotation were ranked in order of TPM, separated in three groups of equal size, and selected randomly in equal proportions (cf. Materials and methods). Among these, specific primers were successfully designed for 18 genes, 11 of which exhibited a band of the expected size (Fig. 3a, Table S3), confirming the transcriptome de novo assembly. Among these, 10 genes with a single band were deemed suitable for RT-qPCR, and the results were compared with RNA-seq coverage. The results were confirmed for 5/10 genes in the TR method, and 9/10 genes in the CT and CC methods (Fig. 3b, c).

## Discussion

### Limitations of RIN in plant transcriptomics

Bioanalyzer RNA Integrity Number (RIN) is an approach that is broadly used for assessing RNA degradation (Mueller et al. 2016; Wang et al. 2016). However, RIN relies on the amount of ribosomal RNAs (18S and 25S for plant RNAs), which means this metric accurately captures the integrity of ribosomal RNAs, but not mRNA integrity (Wang et al. 2016). Therefore, in this study, we used RNA Integrity Number (RIN), transcriptome quality, and differential expression analysis to evaluate several storage and onsite extraction protocols.

An important issue to be aware of when studying non-model plants is that RIN is optimized for model organisms such as humans, and mice because the training data was based on those organisms (Mueller et al. 2016). However, plant cells have additional RNA bands from chloroplast ribosomes, the number and intensity of which may vary by plant species, resulting in an underestimation of the RIN values (Kim et al. 2014). For this reason, TRIzol RNA extraction (T) on samples ground in liquid nitrogen with mortar and pestle was considered to give the maximum possible RIN for this plant. Therefore, in this study, it is assumed the highest quality transcriptome for *H. orientalis* is 6.5–7.7 (Table 3), and this condition was used as a reference to measure the effect of the field-friendly RNA preservation and extraction protocols. RIN numbers within this range are quite common for plants, as shown by a survey of over 600 plant species RNA extraction (Johnson et al. 2012).

Our results indicate that storage for 2 weeks at ambient temperature in either RNA*later* or TRIzol resulted in similar de novo assembly BUSCO completeness scores even for vastly different RIN values (5.2 and 2.3, respectively). On the other hand, storage for 2 weeks in RNA*later* and onsite CC methods showed very different BUSCO scores even though they had the same RIN value (5.2, Table 2). These results serve as a reminder that the RIN is an indicator, but not an absolute guarantee of transcriptome quality, and that proper evaluation through transcriptome analysis is absolutely necessary to conclude about storage or extraction methods. Consistent with our results, Gallelo Romero et al. (2014), using poly(A)-enriched mRNA as we did, and Lu et al. (2022), using rRNA-depletion methods, both conclude that RIN values down to around 4 may be reasonably used for quantifying mRNA expression in human tissue left at room temperature before extraction. However, our results after preserving plant tissue for 14 days in RNA*later* showed a significant decrease in transcriptome quality even though the RIN number was reasonable by those standards (RIN 5.2, Table 2). The ratio of 25S to 18S rRNA is a major influence in RIN estimation (Wang et al. 2016), suggesting the possibility that RNA*later* might preserve ribosomal RNAs better than other transcripts, and we suggest that researchers proceed with caution, as previous studies have shown that the degradation rate may vary significantly between different transcripts (Fleige and Pfaffl 2006; Gallego Romero et al. 2014; Góngora-Castillo and Buell 2013).

### Effect of various tissue storage and RNA extraction methods on the transcriptome

Preservation at room temperature (25 °C) is generally understood to correlate with decreasing RNA integrity (Gallelo Romero et al. 2014; Lu et al. 2022). Preservation of whole cacao beans at room temperature for one week after freeze drying showed similar RNA integrity to that of preservation at − 20 °C for one week, but both were worse than storage at − 80 °C, highlighting − 80 °C as the gold standard for RNA preservation of the plant tissue for RNA extraction (De Wever et al. 2020).

RNA*later* has become a standard preservation solution as an effective alternative to snap freezing in liquid nitrogen in spaceflight, deep sea exploration, isolated areas, and many more (Ghorokova 2005; Hamim et al. 2022; Paul et al. 2005; Yan et al. 2022; Yockteng et al. 2013). For example, Paul et al. (2005) reported successful RNA extraction from Arabidopsis and wheat tissue preserved in RNA*later* for 5 days at 23 °C in spaceflight experiments, but did not report on any transcriptomics results. Hamim et al. (2022) also preserved papaya leaf samples in RNA*later* and successfully detected RNA viruses by RT-PCR after 30 days at room temperature, showing the usefulness of RNA*later* for specific applications. To the best of our knowledge, a full transcriptomics evaluation of the effect of RNA*later* in plants is only available for Arabidopsis leaf tissue stored in RNA*later* at room temperature for 12 h (Kruse et al. 2017). Their results show that RNA*later* affects the response to water, and the hyperosmotic salinity response, among others (> 2.5 log fold change, (*p* ≤ 0.05)), but without distinguishing between up- or down-regulation. Our results similarly show downregulation of the response to acid chemical (*P* < 0.01), salt (*P* < 0.01), and water (*P* < 0.01) after 24 h in RNA*later*, but not at 2 weeks, suggesting that this response is transient. However, photosynthesis was strongly induced by RNAlater in our study, even though they were stored in the dark, suggesting that this response might have been initiated during scissor cutting (Fig. S4). Although preservation in TRIzol was not reported for plant tissue, it has been used as an alternative preservation solution in echinoderm and human tissue, albeit in combination with freezing (Ma et al. 2010; Pérez‐Portela et al. 2013). Our results suggest that RNA*later* and TRIzol may be useful for short fieldwork durations of less than 24 h, but are not applicable to 14-day storage due to low transcriptome completeness and a higher ratio of differentially expressed genes ratio relative to their respective controls (Table 4 and Fig. 2b–d). For 24 h preservation methods (R1 and T1), RNA*later* showed better results in the case of RNA quality and transcriptome quality scores while TRIzol performed better in terms of differential expression analysis and r-square correlation with the control (Table 4 and Fig. 2b–d). Although it is technically possible to recover RNA of relatively decent quality from samples preserved at room temperature for one day, our study confirms that one must consider whether the purpose of the experiment does not conflict with the function of the genes differentially affected by the storage method.

RNA degraded rather quickly (RIN dropped from 7.5 to 5.5 after 24 h, and to 2.1 after 2 weeks) when the tissue was stored in TRIzol at room temperature. Theoretically, no cells are expected to survive the grinding in liquid nitrogen and TRIzol treatment. Therefore, any observed changes should be due to RNA degradation exclusively. However, some upregulated genes are observed (Fig. 2c, d). This may be due to artifacts introduced by the misassembly of fragmented (degraded) RNA during transcriptome assembly and/or differences in stability between different RNAs (Góngora-Castillo and Buell 2013). The resulting loss of diversity in the sequences may also introduce a bias during sequencing, leading to the observed scattered results for lower-expressed genes. These observations are supported by the GO enrichment analysis showing no enrichment of the upregulated genes in terms of function. TRIzol storage methods resulted in an enrichment in downregulated genes of intracellular protein transport (*P* < 0.01) and the regulation of cellular respiration (*P* < 0.01) after 24 h, and protein phosphorylation at 2 weeks (*P* < 1 × 10^–5^), suggesting that those genes may be particularly affected by degradation in TRIzol (Fig. S4). As experimental validation, genes were selected over a range of expression levels and confirmed by RT-PCR, confirming the validity of the de novo assembly. The gene expression levels in TR, CT, and CC were also confirmed with a relatively high success rate for genes in the mid to high expression range, consistent with the higher variability typical of RNA-seq data for the lower range of gene expression.

The results shown here for RNA*later* and TRIzol storage show that the chemical preservation in TRIzol and RNA*later* for two weeks is not feasible at room temperature for transcriptomics applications. Therefore, with long fieldwork durations in mind, we evaluated three different field-friendly RNA extraction protocols using portable equipment including a coffee blender.

Previous attempts at onsite RNA extraction from plants have been made, with mitigated results. Breitler et al. (2016) optimized RNAzol “room temperature” (RT) on tropical crops, a single-step method for RNA extraction in the field, but did not indicate the conditions for shipping to the laboratory. Although they mention the “use of field extracted RNAs in different transcriptomic analyses”, no transcriptome results were shown, apart from a brief mention that the “samples had a quality corresponding to sequencing platform quality standards”, and a real-time PCR (qRT-PCR) evaluation for a single gene was shown instead. It is a well-known fact that qRT-PCR can be successful on partially degraded RNA, as it only amplifies a short fragment (50–150 nt) (Fleige and Pfaffl 2006). Another paper similarly shows the read quality plots coming straight out of the next-generation sequencer, but without showing any further transcriptomics analysis results (Puchta et al. 2020). Needless to say, the quality of the reads themselves is certainly important, but tell us nothing about the transcriptome assembly or gene expression analysis.

Okamura et al. (2020) completed field RNA extraction from soybean roots using the gold standard method (snap-freezing in liquid nitrogen and − 80 °C storage) in the field, which is not feasible in some locations. Furthermore, all of the studies mentioned above make use of kits that contain guanidinium isothiocyanate. Ghawana et al. (2011) and Liu et al. (2018) confirm that guanidinium-based RNA extraction kits are not applicable in plants rich in secondary metabolites, such as the polyphenol and polysaccharide-rich wild strawberry *Fragaria vesca*. Similarly, in our hands, *F. vesca* RNA was totally degraded when using TRIzol control, TR, and CT methods, but not the CC method (Fig. S5 and Fig. S6). Therefore, the CC method is both field-friendly and likely compatible with more plant species.

Although the CTAB extraction method with chloroform phase separation (CC) was slightly more degraded than other methods when compared with the TRIzol laboratory control, transcriptome quality and differential expression ratio showed similar results, which suggests that all three methods can be used as an alternative with few limitations. While TR was the most similar protocol to the control, it changed the transcriptome by 18.75%, with the main contribution being the downregulation of translation. This was observed for all other room temperature extraction methods (Fig. 2c), meaning that performing all extraction steps at room temperature is likely to affect the RNA coding for the factors involved in protein translation. This group of genes are known to respond very quickly to external stress, within a few minutes (Nguyen et al. 2023), suggesting that the efficacy of lysis steps carried out at ambient temperature may be critical. However, the TR method lysis step was done by grinding with mortar and pestle in liquid nitrogen just like the control, the only difference being that subsequent incubation and centrifugation steps were carried out at room temperature, suggesting other causes. From the viewpoint of practicality in the field, the CT and CC methods offer a coffee blender as an alternative for homogenization (Table 1). Interestingly, CT and CC both upregulated photosynthesis (Fig. S4). This upregulation may have happened during the tissue mixing step with the extraction buffer in the blender (Fig. S2). RNA*later* also showed upregulation in photosynthesis, possibly due to leaf-cutting by scissors. However, it seems that coffee bean blender methods influence photosystem II, while RNA*later* influences photosystem I (Fig. S4).

In this study, the coffee bean blender is chosen as an alternative to liquid nitrogen grinding with mortar and pestle. However, RNA purity for the experiments was not ideal, perhaps due to the type of plastic and other materials in the blender cup (Table 2). In addition, some of the upregulation described above might be explained by incomplete homogenization by the blender’s spinning blade (Fig. 2c, d, Fig. S1). Therefore, our next goal is to find better alternatives for homogenization, such as opting for a burr or blade grinder, or grinding the plant tissues directly in CTAB buffer using a hand-held homogenizer or a mortar and pestle as described previously (Doyle and Doyle 1987). Since past studies only report on nucleic acid yield and purity, the effect of homogenization on the transcriptome of various plants remains unknown.

In the case of the TRIzol preservation method and the TRIzol room temperature onsite extraction method (TR), we expect that these differential expression results would be similar in all other plant leaf tissues because the homogenization steps were done by state-of-art liquid nitrogen grinding. In the case of RNA*later* preservation and coffee blender onsite methods, we hypothesize that the differentially expressed genes may vary, especially the upregulated genes, as a function of leaf shape, texture, and/or hardness. Although we did not measure hardness, it took 30–40 s to homogenize *H. orientalis* leaves using the coffee blender (Fig. S2), and unlike strawberry and Arabidopsis leaves, it was not possible to homogenize them with a plastic pestle in a 1.5 mL tube, suggesting that *H. orientalis* are empirically harder. Therefore, we expect to see better results using plants with softer leaf tissues for these protocols. For the harder leaves and tissues, we look forward to further studies investigating other onsite methods by transcriptome analysis.

### Practical implications in the field

What happens after RNA extraction in the field? We suggest that DNase digestion and reverse transcription can be done in the field after extraction, hypothesizing that cDNA would be stable at ambient temperature for at least two weeks, which would make the transportation of the samples possible from remote areas.

However, simply reverse transcribing to cDNA may be enough for qRT-PCR, but not for transcriptome analysis. The preparation of RNA libraries for next-generation sequencing is time-consuming (6–7 h for Illumina, 2 h for Nanopore), on top of the initial DNase digestion step (1 h). Since the RNA extraction itself already requires several hours, it remains very challenging to achieve the whole procedure in the field, and the question of RNA storage is an important one. Breitler et al. (2016) suggested that RNA pellets that are preserved in ethanol for up to 5 days can be used for transcriptome analysis, without providing evidence. The extracted *H. orientalis* RNA pellets using the onsite CT protocol were stored in 75% Ethanol for 2 weeks at 25 ℃, and the RIN remained the same (Fig. S7), but further transcriptome analysis is needed to fully evaluate the RNA pellet preservation method. Zhang et al. (2015) demonstrated that total urinary RNA dried on nylon membranes and stored in vacuum bags for three months didn’t change the RNA yield and quantification of microRNA (small RNAs which are generally 20–22nt). Puddu et al. (2015) developed a room-temperature RNA preservation technology in silica microcapsules which made RNA as stable as DNA. These techniques may be an alternative for the long-term preservation of pure RNA after the extraction in the field.

DeLTA-Seq: direct-lysate targeted RNA-seq method by Kashina et al. (2022) showed that it is possible to skip the RNA extraction step and directly proceed with reverse transcription from the plant tissue lysate in buffer containing 100 mM DTT/90 mM Tris–HCl (pH7.6) for large-scale experiments using a sample pooling technique. The DeLTA-Seq method was demonstrated in Arabidopsis, rice, and wheat, and is focused on approximately 100 targeted genes of model plants, but requires prior knowledge of the transcriptome to design the primers. Our study focuses on the whole transcriptome of non-model plants, for which prior sequence information is not available. In the future, if it becomes possible to prepare the next-generation sequencing libraries directly on the whole cell extract and totally bypass RNA extraction, this would save precious time in the field.

Even though these onsite protocols need to be tested in more plants and further optimization is needed including reducing the effect of using the coffee blender, with the advantage of not using liquid nitrogen for homogenization and the lysis step, among the methods presented here, the CC method is the most practical and compatible with various plants including those with rich content in secondary metabolites such as polyphenols. We expect this method to be useful in remote areas such as deserts, high mountains, and deep forests, where cold transportation of samples is impractical.

All in all, the transcriptomic analysis of *H. orientalis* with two different preservation methods and three onsite extraction methods showed that all protocols tested resulted in some degree of change in gene expression profiles when compared to their respective controls. Therefore, we strongly recommend carefully choosing the protocol based on a combination of the purpose of the study, including the target gene pathways, and the technical limitations associated with the target fieldwork location (availability of liquid nitrogen, dry ice or cool storage).

## Supplementary Information

Below is the link to the electronic supplementary material.

Supplementary file1 (PDF 3698 KB)

Supplementary file2 (XLSX 2425 KB)

## Data Availability

The data underlying this article is available at the Sequence Read Archive (SRA) database under the BioProject accession: PRJNA1081167 Helonias orientalis Transcriptome.
