# Supplementary material for: Effect of fieldwork-friendly coffee blender-based extraction methods and leaf tissue storage on the transcriptome of non-model plants
Source: J Plant Res. 2025 Mar 7;138(3):511–24. doi: 10.1007/s10265-025-01624-w (PMC12062031; doi:10.1007/s10265-025-01624-w)
Supplement: Supplementary file 1 — Supplementary file1 (PDF 3698 KB) [file 10265_2025_1624_MOESM1_ESM.pdf]

Supplementary material

Effect of fieldwork-friendly coffee blender-based extraction methods and leaf tissue storage on the transcriptome of non-model plants

Journal of Plant Research

Shine Undarga Dagva[1,2], Josephine Galipon[1,2,3]

[1] Present: Graduate School of Science and Engineering, Yamagata University, Yamagata, Yonezawa, Japan;

[2] Institute for Advanced Biosciences, Keio University, Yamagata, Tsuruoka, Japan;

[3] Graduate School of Media and Governance, Keio University, Kanagawa, Fujisawa, Japan

Corresponding author: Josephine Galipon

E-mail address: jgalipon@yz.yamagata-u.ac.jp

1.1 Nucleic acid extraction (DNA+RNA)

T (control): RNA was extracted by TRIzol reagent according to the manufacturer's manual with small modifications.

A fresh leaf was harvested from the pot and ground into powder in liquid N_2 by mortar and pestle. 0.05 g of the ground powder was added to a volume of 1 ml TRIzol solution in an RNase-free 1.5 ml tube. The mixture was vortexed briefly and incubated at room temperature for 5 minutes. 980 µl of the mixture was transported to a new 1.5 ml tube in order to get rid of the plant debris and 200 µl of chloroform was added to the mixture, then vortexed, and incubated at room temperature for 2 minutes. The mixture was centrifuged at 12000 g for 15 minutes at 4 °C. 500 µl of the aqueous phase was carefully transported to a new 1.5 ml tube, 500 µl of isopropanol was added, mixed thoroughly by vortex and incubated at room temperature for 10 minutes. The sample was centrifuged at 12000 g for 10 minutes at 4 °C. The supernatant was removed by a pipette and the pellet was washed by 800 µl of 75% ethanol. The sample was centrifuged at 12000 g for 5 minutes at 4 °C. The supernatant was removed again and the pellet was washed again by 75 % ethanol. The sample was centrifuged again at 12000 g for 5 minutes at 4 °C. The supernatant was discarded and the pellet was dried for 3 min in a vacuum. The pellet was resuspended in 40 – 100 µl of distilled water.

R1 (storage): 0.2 g of plant leaf was cut by sterilized scissors into smaller pieces and collected into 1.3 ml of RNA*later* in a 1.5 ml tube.

After preserving at 25 °C for 24 hours, the leaf particles were briefly dried on sterile paper tissue, and then ground in liquid nitrogen using mortar and pestle. The remaining steps were followed as in the state-of-art method, T.

R14 (storage): 0.2 g of plant leaf was cut by sterilized scissors into smaller pieces and collected into 1.3 ml of RNA*later* in a 1.5 ml tube.

After preserving at 25 °C for 14 days, the leaf particles were briefly dried on sterile paper tissue, and then ground in liquid nitrogen using mortar and pestle. The remaining steps were followed as in the state-of-art method, T.

T1 (storage): A fresh leaf was harvested from the pot and ground into powder in liquid N_2 by mortar and pestle.

0.05 g of the ground powder was preserved in 1 ml of TRIzol solution in an RNase-free 1.5 ml tube at 25 °C for 24 hours. The remaining steps were followed as in the state-of-art method, T.

T14 (storage): A fresh leaf was harvested from the pot and ground into powder in liquid N_2 by mortar and pestle.

0.05 g of the ground powder was preserved in 1 ml of TRIzol solution in an RNase-free 1.5 ml tube at 25 °C for 14 days. The remaining steps were followed as in the state-of-art method, T.

TR (on-site): RNA was extracted by TRIzol reagent according to the manufacturer's manual with few modifications to use with portable equipment.

A fresh leaf was harvested from the pot and ground into powder in liquid N_2 by mortar and pestle. 0.05 g of the ground powder was added to a volume of 1 ml TRIzol solution in an RNase-free 1.5 ml tube. The mixture was vortexed briefly and incubated at room temperature for 5 minutes. 980 µl of the mixture was transported to a new 1.5 ml tube in order to get rid of the plant debris. 200 µl of chloroform was added to the mixture, then vortexed, and incubated at room temperature for 2 minutes. The mixture was centrifuged at 12000 g for 15 minutes at <u>room temperature</u>. 500 µl of the aqueous phase was carefully transported to a new 1.5 ml tube, 500 µl of isopropanol was added, mixed thoroughly by vortex and incubated at room temperature for 10 minutes. The sample was centrifuged at 12000 g for 10 minutes at <u>room temperature</u>. The supernatant was removed by a pipette and the pellet was washed by 800 µl of 75 % ethanol. The sample was centrifuged at 12000 g for 5 minutes at <u>room temperature</u>. The supernatant was removed again and the pellet was washed again by 75 % ethanol. The sample was centrifuged again at 12000 g for 5 minutes at <u>room temperature</u>. The supernatant was discarded and the pellet was dried <u>at 37 °C for 10 minutes</u>. The pellet was resuspended in 40 µl of distilled water.

CT (on-site): CTAB TRIzol, Plant leaf homogenized in CTAB EB using a coffee-blender followed by the addition of TRIzol reagent. The remaining steps are as per the instructions in the TRIzol reagent manual, but at room temperature.

A newly harvested fresh leaf (0.5 g) in 13 ml of CTAB EB and 195 µl of beta mercaptoethanol (1.5 %) was mixed for 40 seconds by mini-blender and incubated at room temperature for 3 minutes. 500 µl of the mixture was transported into an RNase-free 2 ml tube and 1 ml of TRIzol solution was added to the sample and the final mixture was incubated at room temperature for 5 minutes. 200 µl of chloroform was added to the mixture, then vortexed, and incubated at room temperature for 2 minutes. The mixture was centrifuged at 12000 g for 15 minutes at <u>room temperature</u>. 900 µl of the aqueous phase was carefully transported to a new 2 ml tube, 900 µl of isopropanol was added, mixed thoroughly by vortex and incubated at room temperature for 10 minutes. The sample was centrifuged at 12000 g for 10 minutes at <u>room temperature</u>. The supernatant was removed by a pipette and the pellet was washed by 800 µl of 75 % ethanol. The sample was centrifuged at 12000 g for 5 minutes at <u>room temperature</u>. The supernatant was removed again and the pellet was washed again by 75 % ethanol. The sample was centrifuged again at 12000 g for 5 minutes at <u>room temperature</u>. The supernatant was discarded and the pellet was dried <u>at 37 °C for 10 minutes</u>. The pellet was resuspended in 40 µl of distilled water.

CC (on-site): CTAB coffee-blender, Plant leaf homogenized in CTAB EB using a coffee-blender. The remaining steps were carried out as in Gambino et al. (2008), but without lithium chloride precipitation and at room temperature.

A newly harvested fresh leaf (0.5 g) in 13 ml of CTAB EB and 195 µl of beta-mercaptoethanol (1.5 %) was mixed for 40 seconds by mini-blender and incubated at room temperature for 3 minutes. 900 µl of the mixture was transported into an RNase-free 2 ml tube, 900 µl of chloroform was added to the solution, and mixed thoroughly by vortex. The phase separation was done by centrifugation at 12000 g for 5 minutes at <u>room temperature</u>. 800 µl of the aqueous phase was transported into a new 2 ml tube, 800 µl of chloroform was added to the solution, and mixed thoroughly by vortex. The phase separation was done by centrifugation at 12000 g for 5 minutes at room temperature.

500 µl of the aqueous phase was transported into a new 1.5 ml tube, 500 µl of isopropanol was added, mixed thoroughly by vortex and incubated at room temperature for 10 minutes. The sample was centrifuged at 12000 g for 10 minutes at <u>room temperature</u>. The pellet was resuspended in 400 µl of distilled water, 16 µl of ammonium

acetate and 400 µl of isopropanol was added to the sample and incubated at room temperature for 10 minutes. The sample was centrifuged at 12000 g for 10 minutes at room temperature. The supernatant was removed by a pipette and the pellet was washed by 800 µl of 75 % ethanol. The sample was centrifuged at 12000 g for 5 minutes at room temperature. The supernatant was removed again and the pellet was washed again by 75 % ethanol. The sample was centrifuged again at 12000 g for 5 minutes at room temperature. The supernatant was discarded and the pellet was dried at 37 °C for 10 minutes. The pellet was resuspended in 40 µl of distilled water.

1.2 Total RNA extraction

DNase I digestion: All samples were digested by recombinant DNase I (Takara Bio Inc.) according to the manufacturer's instructions with small modifications using Eppendorf MiniSpin® plus. Inactivation of DNase I was done by heat treatment as described in the manual. Resulting pellet was dissolved in MilliQ water.

25 µl of mixture (20 µl of sample, 2.5 µl of 10 x DNase I Buffer, 1 µl of Recombinant DNase I and 1.5 µl of distilled water) was incubated at 37 °C for 20 minutes. 2.5 µl of 0.5 M EDTA was added to the sample and incubated at 80 °C for 2 minutes. The sample volume was increased to 100 µl with RNase-free water, 4 µl of 7.5 M ammonium acetate (final concentration 0.3 M) and 100 µl of isopropanol were added and incubated at room temperature for 10 minutes. The sample was centrifuged at 12000 g for 5 minutes at room temperature. The supernatant was removed and the pellet was washed with 75 % ethanol. The sample was centrifuged at 12000 g for one minute at room temperature. The pellet was dried and re-suspended in 20 µl of distilled water.

Supplementary tables

Table S1. RNA-Seq results of before and after quality control.

Methods	Raw reads*	Clean reads**	Read loss (%)
T	26,666,667	26,619,131	0.18
R1	26,666,667	26,617,381	0.18
R14	26,666,667	26,614,814	0.19
T	26,666,667	26,590,280	0.29
T1	26,666,667	26,612,698	0.20
T14	26,666,667	26,602,120	0.24
T	26,333,334	26,208,624	0.47
TR	26,333,334	26,205,648	0.48
CT	26,333,334	26,256,055	0.29
CC	26,333,334	26,255,060	0.29

*The total raw data were downsampled by random selection using seqtk to approximately 8 Gb for each sample (Li 2012).
**Clean paired reads were trimmed by using Trim Galore version 0.6.7 (Phred score of Q = 20, stringency 6 bp, removal of reads below 100 bp as well as non-paired reads) (Felix et al. 2023).

Table S2. DEG functional annotation, GO enrichment, and fold-change values

Excel file “GO_enriched_DEGs.xlsx

Table S3. Selection of transcripts and the primers for RT-qPCR

Label	Transcript Name	GO number	GO meaning	Primers		Expected Band Size
Gene A	TRINITY_DN8584_c0_g1_i10	GO:0005576	extracellular region	Forward	GTCGTTGTTGGGGAGGTCAT	183 bp
				Reverse	GTACAGCGAAGGATACGGGG	
Gene B	TRINITY_DN22707_c0_g3_i1	GO:0003676| GO:0008270	nucleic acid binding, zinc ion binding	Forward	CCCCGACGTGAATTGCATTC	123 bp
				Reverse	CGTGTCCATGACGGCTATGA	
Gene C	TRINITY_DN69006_c0_g1_i1	GO:0009408| GO:0010115	response to heat, regulation of abscisic acid biosynthetic process	Forward	CGACGCTGCACAGAAATACG	191 bp
				Reverse	TCTCCTTCAACAACCGGCTC	
Gene D	TRINITY_DN21064_c0_g1_i2	GO:0003676| GO:0004523	nucleic acid binding, RNA-DNA hybrid ribonuclease activity	Forward	TGACCTGGGGATCAAGCAAC	64 bp
				Reverse	GCGAGTGCGTCTGCTAGTAT	
Gene E	TRINITY_DN42639_c0_g1_i6<br>TRINITY_DN42639_c0_g1_i1<br>TRINITY_DN42639_c0_g1_i16<br>TRINITY_DN42639_c0_g1_i18	GO:0004674| GO:0005524	protein serine/threonine kinase activity, ATP binding	Forward	CGCATCGATCATTGCAGCAA	153 bp
				Reverse	AGACTCAACAGTGATGCCCG	
Gene F	TRINITY_DN13262_c0_g1_i24	GO:0005515	protein binding	Forward	GAAAGGGGTATGCTGCCCTT	167 bp
				Reverse	TAATCGCTTCGAGGGTCCAC	
Gene G	TRINITY_DN5050_c1_g2_i8	GO:0003676	nucleic acid binding	Forward	GATGCGACGACCAAATTGGG	90 bp
				Reverse	TTGGGTGTTTTTGCTGTGCC	
Gene H	TRINITY_DN1560_c0_g1_i4	GO:0000166| GO:0004832| GO:0005524| GO:0006438	nucleotide binding, valine-tRNA ligase activity, ATP binding, valyl-tRNA aminoacylation	Forward	TGTCCTGCATGAAGCTCAGG	188 bp
				Reverse	TCCCAGCCTACAAACAGCAG	
Gene I	TRINITY_DN78805_c0_g1_i1	GO:0005881| GO:0016020	cytoplasmic microtubule, membrane	Forward	GGCCCTTCTGTTCCTCAGTC	98 bp
				Reverse	TTGAATGGCTGCTCACCGAT	
Gene J	TRINITY_DN87928_c0_g1_i1	GO:0003676	nucleic acid binding	Forward	TCGGAGGTCGACCAGATTCT	156 bp
				Reverse	CAGCTCTTCTGCGAAGTCCA	
Gene K	TRINITY_DN18789_c0_g1_i5	GO:0005515	protein binding	Forward	TGCAGCACCTCTTGTTCACA	182 bp
				Reverse	TGGGAGAGAGGAAGAGAGCC	

Gene L	TRINITY_DN1961_c0_g1_i9	GO:0003700| GO:0006355| GO:0043565	DNA-binding transcription factor activity, regulation of DNA-templated transcription, sequence-specific DNA binding	Forward	GCTGGTAGATCAGGTGCTCC	102 bp
				Reverse	ATGGCCGTTGATCTGATGGG	
Gene M	TRINITY_DN2069_c0_g1_i1	GO:0005507| GO:0005743| GO:0006878| GO:0008535| GO:0016531	copper ion binding, mitochondrial inner membrane, intracellular copper ion homeostasis, respiratory chain complex IV assembly, copper chaperone activity	Forward	GAATTCCATCGCTGGGTCCA	193 bp
				Reverse	ATTCCGTCGAGCAAGTTCGT	
Gene N	TRINITY_DN1325_c0_g3_i1	GO:0006979| GO:0016671| GO:0030091	response to oxidative stress, oxidoreductase activity, acting on a sulfur group of donors, disulfide as acceptor, protein repair	Forward	AGCCAGAAGAAGCCATGGTC	111 bp
				Reverse	CCTGGTTGGATTTCCGTCCA	
Gene O	TRINITY_DN13281_c0_g3_i1	GO:0016787	hydrolase activity	Forward	TCTCGGTGGTTCTTCCTTGC	120 bp
				Reverse	GCCCGAGAGAGAATAGGCAC	
Gene P	TRINITY_DN56702_c0_g2_i1	GO:0003824	catalytic activity	Forward	AGATTGCCAAGGCTCAGGTC	118 bp
				Reverse	GTCATGGAAAGCAAGGCACC	
Gene Q	TRINITY_DN126055_c0_g1_i1	GO:0010309	acireductone dioxygenase [iron(II)-requiring] activity	Forward	CTGGTATATGGACGACGCCC	111 bp
				Reverse	CAGCTTCCAGTAGAGCACCC	
Gene R	TRINITY_DN137464_c0_g1_i1	GO:0098609	cell-cell adhesion	Forward	CAGGCTGAGATTGGGCAGAA	182 bp
				Reverse	CTTCTTGGCCCCAAAGCAAC	

Supplementary figures

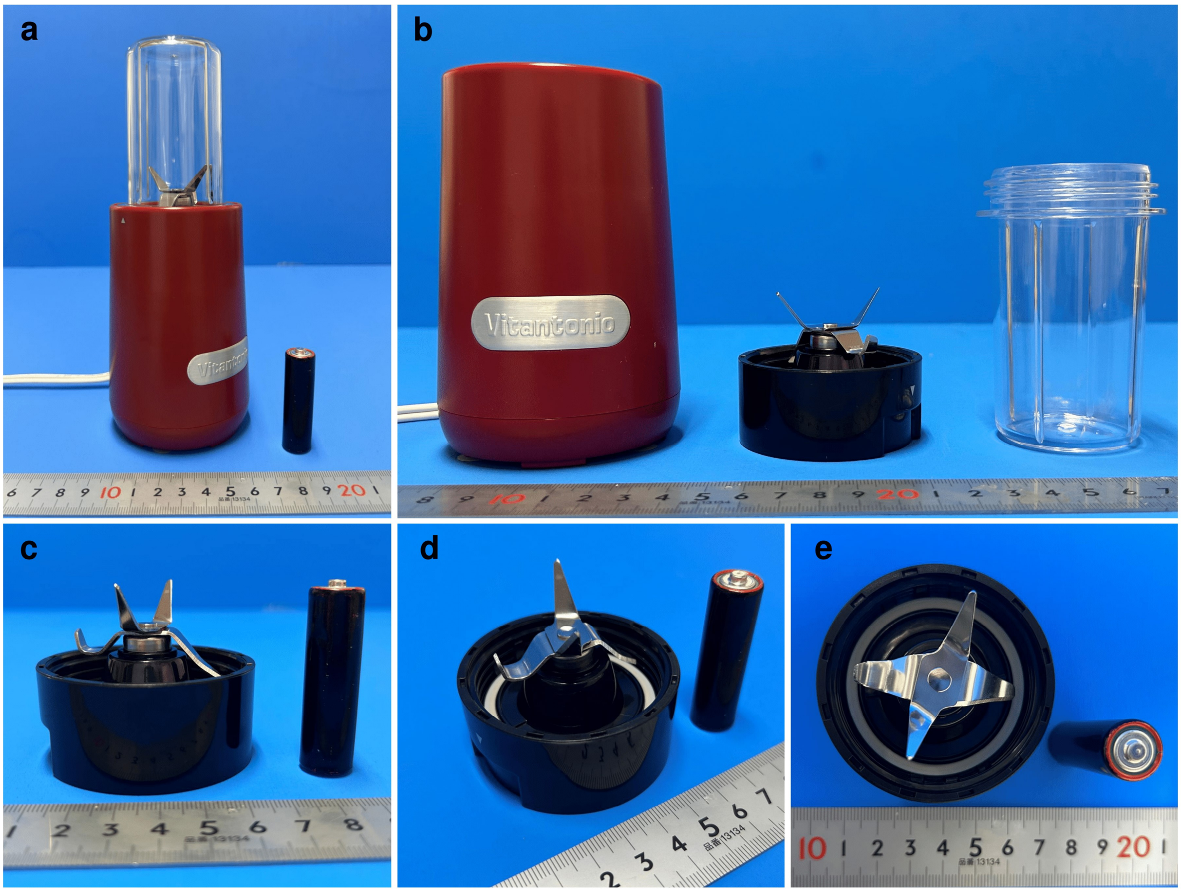

Fig. S1. Coffee blender Vitantonio Mini Bottle Blender VBL-6 used in this study. **a**. Whole side view **b**. Side view separated **c**. Isometric view of the blade **d**. Side view of the blade **e**. Top view of the blade.

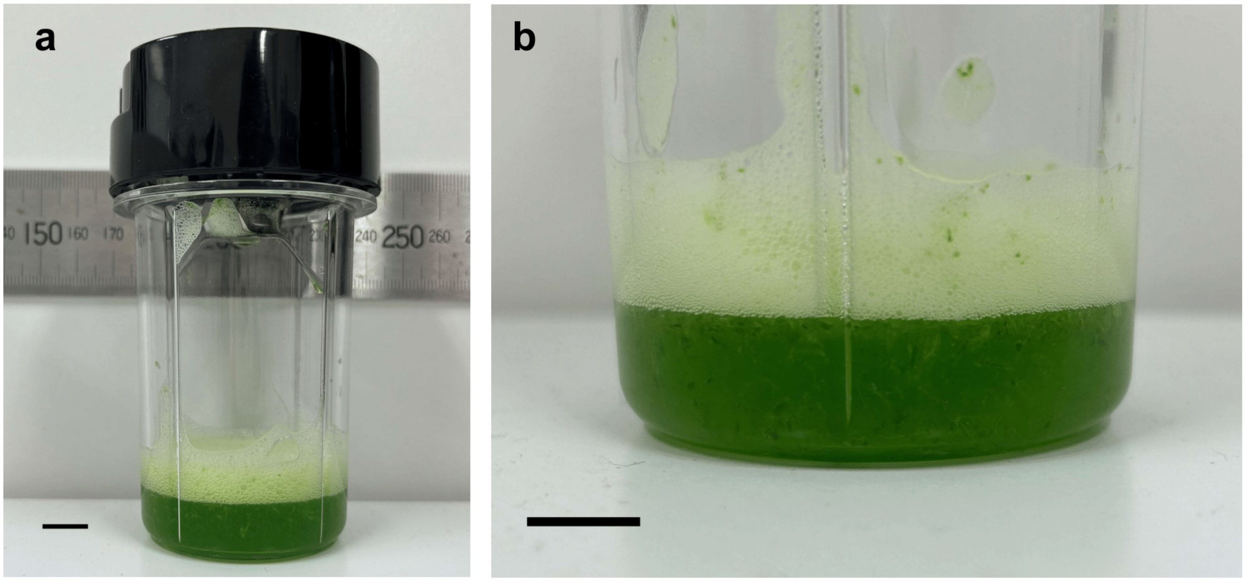

Fig. S2. Plant tissue homogenized in CTAB by coffee-bean blender for 40 seconds. **a**. Whole view. **b**. Zoomed in view. The scale bar is 1 cm.

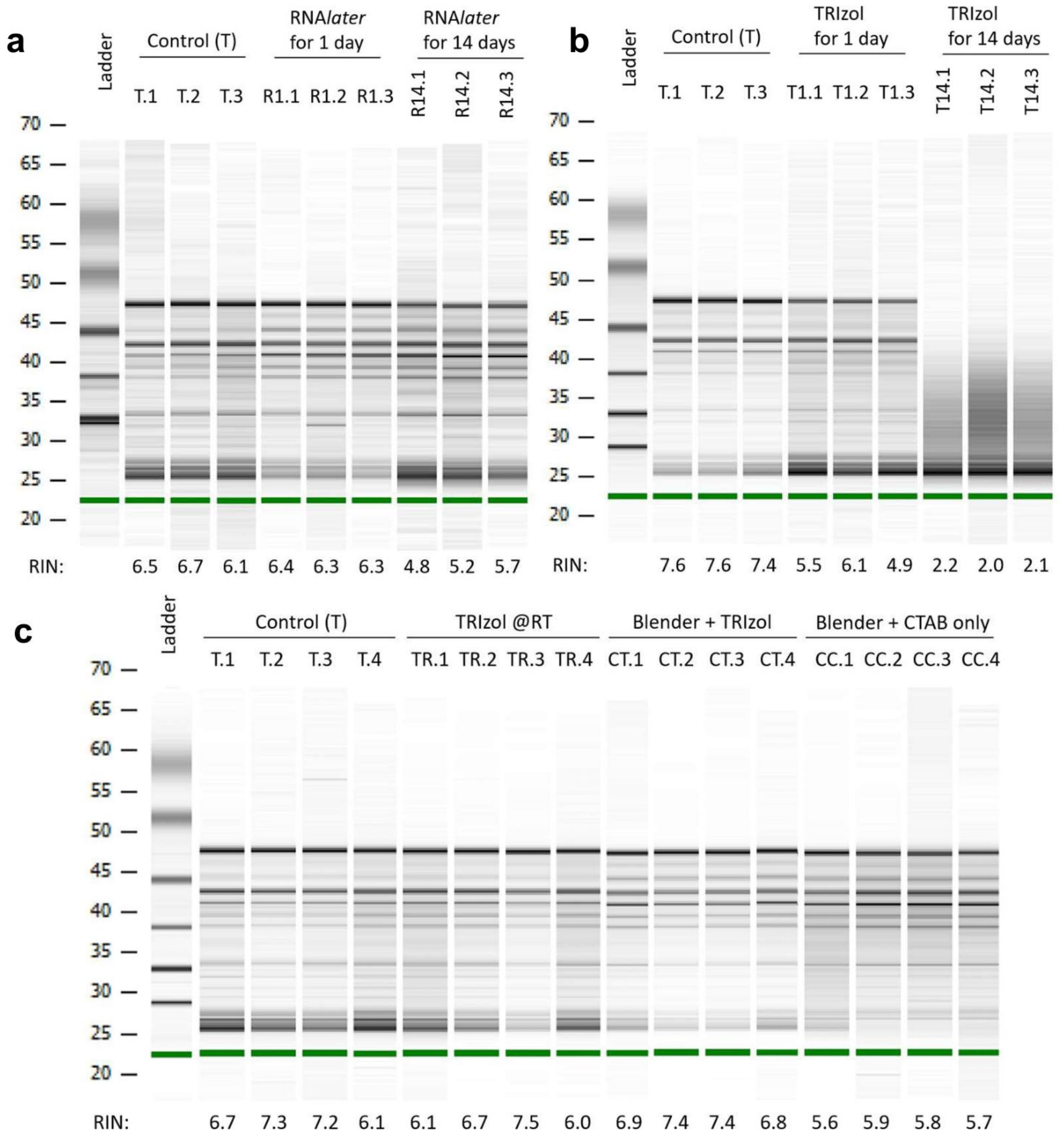

Fig. S3. Electrophoresis and RIN values for all tested conditions and replicates with *H.orientalis*. **a**. Storage in RNA*later* (n = 3). **b**. Storage in TRIzol (n=3). c. onsite extraction protocols (n = 4) with their control (T). All samples were measured with the RNA 6000 Nano Kit on an Agilent 2100 Bioanalyzer. The numbers after dots represent the replicate number.

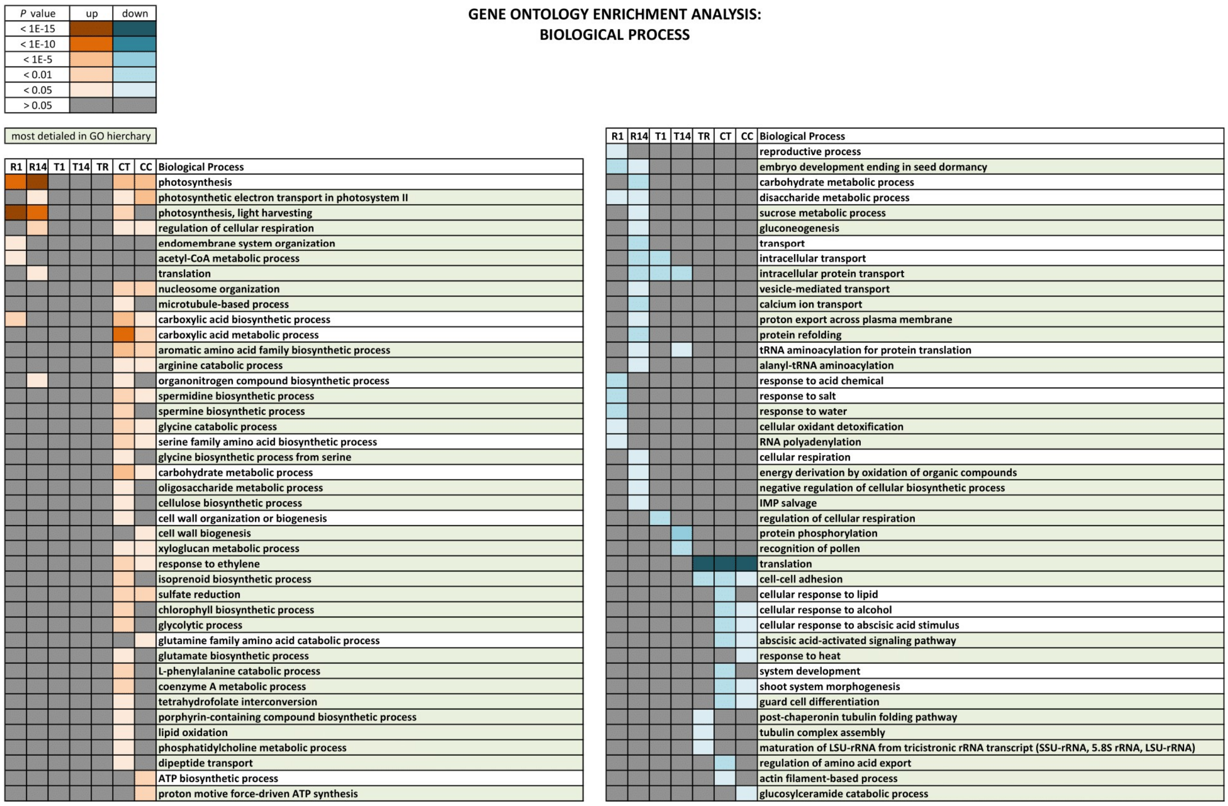

Fig. S4. Simplified result of Biological Process by Gene Ontology Enrichment Analysis using the BiNGO tool in Cytoscape, showing the overrepresentation of upregulated (orange) and downregulated (blue) genes. The statistical significance of the enrichment is indicated as a color gradient (cf. top-left legend).

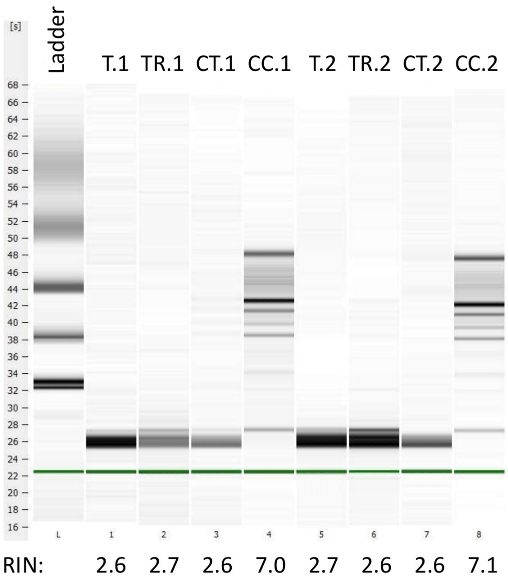

Fig. S5. Electrophoresis and RIN values for for all tested conditions with *Fragaria vesca*. T: control, onsite TR: TRIzol at room temperature, CT: CTAB in coffee bean blender followed by TRIzol, CC: CTAB in coffee bean blender. All samples were measured using the RNA 6000 Nano Kit on an Agilent 2100 Bioanalyzer. These conditions were replicated (n = 4), of which only two were checked by Bioanalyzer. Due to the high level of degradation when using TRIzol, the experiment was stopped at this stage. The numbers after dots represent the replicate number.

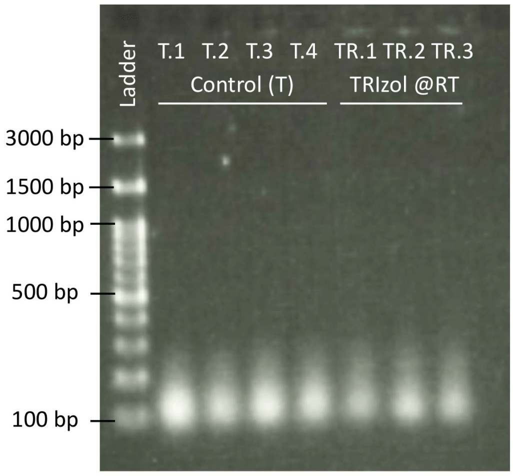

Fig. S6. Electrophoresis image of control (T) (n = 4), onsite TR: TRIzol at room temperature protocols (n = 3) of *Fragaria vesca* leaves, on a 1.5 % TBE gel stained with ethidium bromide.

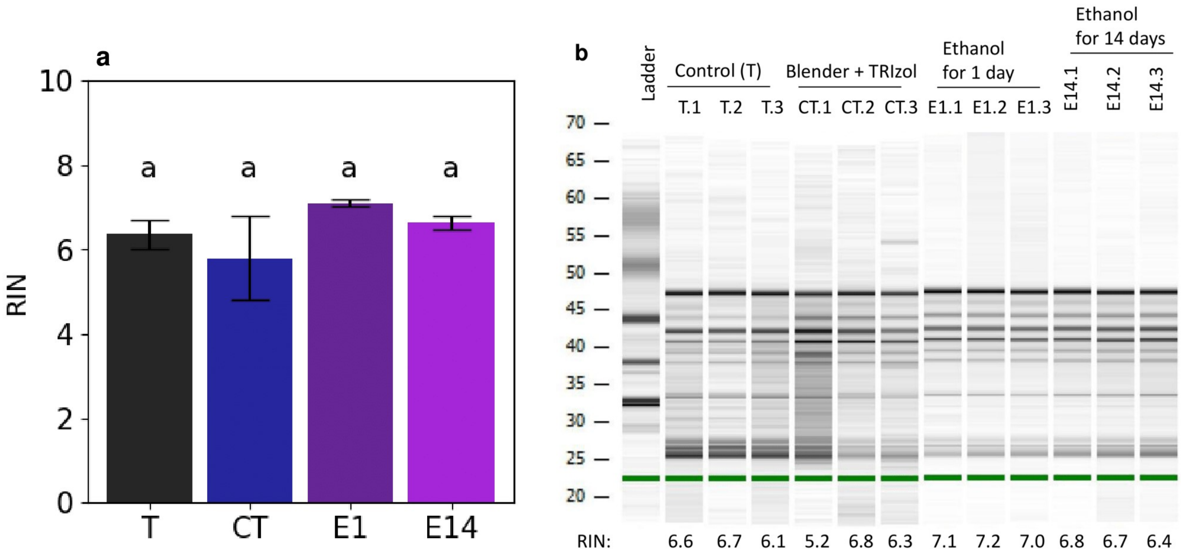

Fig. S7. RNA quality of RNA extracted from *H. orientalis* leaves measured by Agilent 2100 Bioanalyzer using the Agilent RNA 6000 Nano Kit **a**. Bar graph of the RNA quality (RIN). T: TRIzol control, CT: CTAB in coffee bean blender followed by TRIzol onsite extraction method, E1: Extraction by CT method, followed by storage of the pellet in the 75 % ethanol of the last wash step for 1 day at 25 ºC, E14: the same as for E1 but stored for 14 days at 25 ºC. All samples were subjected to DNase digestion before running on the Bioanalyzer. The results were compared using the ANOVA post hoc Tukey Honestly Significant Difference Test ($P < 0.05$). Different letters show significant differences, therefore there were no significant differences between any of the protocols. **b**. Electrophoresis by Bioanalyzer of all samples. All extractions were replicated three times ($n = 3$).
